# Towards the Automatic Scanning of Indoors with Robots

**DOI:** 10.3390/s150511551

**Published:** 2015-05-19

**Authors:** Antonio Adán, Blanca Quintana, Andres S. Vázquez, Alberto Olivares, Eduardo Parra, Samuel Prieto

**Affiliations:** 3D Visual Computing and Robotics Lab, Universidad de Castilla-La Mancha (UCLM), Paseo de la Universidad, 4, Ciudad Real 13071, Spain; E-Mails: Blanca.Quintana@uclm.es (B.Q.); andress.vazquez@uclm.es (A.S.V.); alberto.olivares@alu.uclm.es (A.O.); Eduardo.Parra1@alu.uclm.es (E.P.); Samuel.Prieto@uclm.es (S.P.)

**Keywords:** indoor scanning, LiDAR sensors, BIM, automatic 3D digitization

## Abstract

This paper is framed in both 3D digitization and 3D data intelligent processing research fields. Our objective is focused on developing a set of techniques for the automatic creation of simple three-dimensional indoor models with mobile robots. The document presents the principal steps of the process, the experimental setup and the results achieved. We distinguish between the stages concerning intelligent data acquisition and 3D data processing. This paper is focused on the first stage. We show how the mobile robot, which carries a 3D scanner, is able to, on the one hand, make decisions about the next best scanner position and, on the other hand, navigate autonomously in the scene with the help of the data collected from earlier scans. After this stage, millions of 3D data are converted into a simplified 3D indoor model. The robot imposes a stopping criterion when the whole point cloud covers the essential parts of the scene. This system has been tested under real conditions indoors with promising results. The future is addressed to extend the method in much more complex and larger scenarios.

## 1. Introduction

Today, there are multiple devices and applications that have introduced the 3D component in different sectors, such as architecture, engineering and construction (AEC). In the AEC context, applications go from reverse engineering using 3D scanners and 3D printers to 3D building digitization and implementation of the model in a film or a video game. Areas, like medical and cultural, and many others, have also benefited from the 3D digitization; examples include the possibility of prosthesis construction adapted to the anthropometry of each patient or the possibility of making virtual tours through historic buildings.

3D scanners have improved over the years in precision and range, building digitization becoming one of the fields that makes greater use of this technology. 3D digitization is frequently used to carry out quality and precision controls in the construction of facilities. Throughout a CAD (Computer-Aided Design) model obtained from the point clouds provided by the scanners, the engineers can recover non-existent models or evaluate deviations or faults in buildings with respect to the existing planes.

In the automatic digitization world, we first have to distinguish between automatic scanning and automatic modeling. In this paper, we only focus on the first subject: automatic scanning. Specifically, the objective of this paper is to present a methodology that carries out a complete scanning process of the interiors of buildings using a 3D laser scanner on board a mobile robot.

For many years, the 3D digitization of buildings and large-scale environments has been carried out manually by an operator. Some good examples of this can be found in [1], where the 3D digitization of a Chinese city is done, [2], which presents the digitization of the Acropolis of Athens, and [3], where the 3D digitization process of different buildings is explained. Despite the fact that manual digitization is currently a practice common in this area, however, it has some serious problems mainly concerning the arbitrary number of scans taken, the time and the precision of the scans. Such problems can be reduced by automating a part of or the whole scanning process. Thus, automatic scanning has become one of the most exciting 3D computer vision research lines in the last few years.

Other approaches perform permanent scans in cars, on board humans or even with mobile robots. The key point here is that most of those SLAM (Simultaneous Localization And Mapping) systems are manually commanded. Therefore, a human decides where to go and how to perform a complete scanning of a large scenario. This is clear in the case of cars and humans carrying scanners. For example, in Toschi *et al.* [4], a laser scanning system on board a chauffeur-driven car digitizes the downtown of Trento. Xiao *et al.* [5] reconstruct indoors by means of an RGB-D camera that is handled by a person. The individual who carries the system takes care of avoiding data redundancy while he moves. A robot with a 3D scanner is used in [6]. Curiously, the robot is used just to facilitate the registration of scans taken, but it is manually teleoperated. Micro-aerial vehicles (MAV) even can extract 3D information on interior scenes, like in [7]. The authors propose an indoor mapping approach using a MAV. The MAV is equipped with a camera and a laser range finder that takes data from different heights in the room. To integrate the scans, those systems use, among other technologies, timestamps and IMUs (Inertial Measurement Unit).

As regards autonomous robots, many authors use 2D laser scanners for navigation and reconstruction of 2D maps, but few papers address automatic 3D digitization of interiors. Some of the works close to ours are included in this section.

In order to automate the 3D digitization process, one of the essential points is to automatically decide the next scanner position. The decision of the best next positions should lead us to a complete, quality and non-time-consuming digitization of the scene. This is known in the literature as the next best view problem (NBV).

The original NBV's concept was discussed by Conolly *et al.* in [8] and applied for object reconstruction. From then, several works have been devoted to introduce NBV solutions in the building and facilities context using 3D data. A 3D NBV algorithm for the reconstruction of indoor environments with polynomial functions in a voxelized space is proposed in [9]. An extended version of this is introduced by Lopez–Damian *et al.* by using probabilistic functions [10]. They evaluated the efficiency of their algorithms on simulated indoor scenes. Low *et al.* present a novel strategy based on hierarchical NBVs [11] in which the model of the scene is represented in an octree space. Another space representation is adopted in [12] with the aim of solving the NBV problem efficiently. In this case, a 2D grid of cells stores different 3D attributes of the points projected in each cell. The stored information is about the minimal and maximal height of the projected points, if the cell contains scan points higher or lower than the exploration platform height, if the cell belongs to a wall or the ground, and so on. The use of this grid of cells reduces the amount of data compared with similar 3D grid structures, thus reducing the computation time.

Beyond methods dealing with the NBV problem, we are interested in complete and automatic digitization solutions. The fact is that there are few articles that address the problem of automatic digitization, and therefore, they use a mobile device that autonomously navigates and takes scans of the environment until the scene is completely sensed. Some of the scarce proposals of this automatic scanning are as follows.

Sanchiz *et al.* [13] present an NBV algorithm for automatic reconstruction of 3D scenes with a 3D sensor on a pan/tilt head. They simulated the method on a simple scene, but the fact that this is one of the first articles that developed the automatic reconstruction of 3D environments is taken into consideration. Surmann *et al.* [14] extend a previous 2D NBV algorithm proposed by Gonzalez *et al.* [15]. They use a scanner on board a mobile robot and take several slices of points. Then, they apply a 2D NBV approach over all of the slices and choose the best solution. In reality, the experiments demonstrate that a unique slice is sufficient in most of the cases.

Semiautomatic methods can also be found, as the one of Blaer *et al.* in [16]. They present an algorithm for digitizing outdoor areas also using a mobile robot and a 3D scanner, but in this case, the system needs an initial 2D map of the environment to be scanned. The same algorithm has been used in [17] as a part of a more complex process in which the recognition of rooms is introduced. They combine 2D exploration and 3D NBV algorithms. Another interesting work is found in [18]. The authors present an NBV algorithm for kitchen reconstruction, which evaluates the entropy from different scanner positions and takes as the NBV position the one with the highest information gain. The same idea about information gain is applied in [19]. In this case, the NBV algorithm is defined by using the probability of Markovian observability.

As was said before, to achieve a precise 3D model of the scene, further 3D data processing tasks are necessary. In this case, the objective is to process the huge unstructured point cloud and to generate a simplified 3D CAD-like model of the scenario in an automatic manner. This process is called automatic construction of basic BIM (building information modeling) models. A review about different techniques for BIM model reconstruction can be found in [20]. Below, we make a brief reference to the most relevant works in this area and that are close to our current research.

There are some approaches that segment the scene into different essential and structural parts of the building. This is the case presented in [21], where the authors segment the point cloud and extract the points belonging to the walls, floor and ceiling of a room. They dealt with the occlusion problem and identify the occluded regions in walls and floors. Besides, openings are also delimited. Mura *et al.* [22] present a method with a similar objective, but without detecting either occlusion or openings in the walls. Another works in this line can be found in [23,24]. In [25], an automatic reconstruction of a semantic 3D model of inhabited indoors is carried out with the help of RFID (Radio Frequency Identification) technologies. They segment the structural parts of the room, like walls, ceiling, floor, windows and doors, and also identify a set of basic movable objects, like tables, chairs and cabinets. Basic BIM models in buildings have also been generated in [26,27].

As we can see, there are few articles in which a basic model of a real environment is generated that has been previously digitized autonomously with a mobile robot. One of the scarce works that achieves a total automation is the one of the Blodow *et al.* [18]. In this, a semantic model of a kitchen is built after digitizing the scene with a mobile robot. The semantic model is used by the robot to interact with pieces of furniture, being able to open and close doors and drawers.

The paper is organized as follows. Section 2 presents an overview of our automatic digitization strategy. Section 3 is devoted to explaining the main processes carried out in the first scan, including the alignment and filtering of the indoor point cloud and the obtaining of the first robot map. The NBV algorithm is presented in Section 4, whereas Section 5 deals with the treatment of the following scans. Experimental results are detailed in Section 6. Finally, in Section 7, we discuss the conclusions, contributions and future proposals.

## 2. An Overview of the Method

**Figure 1:**
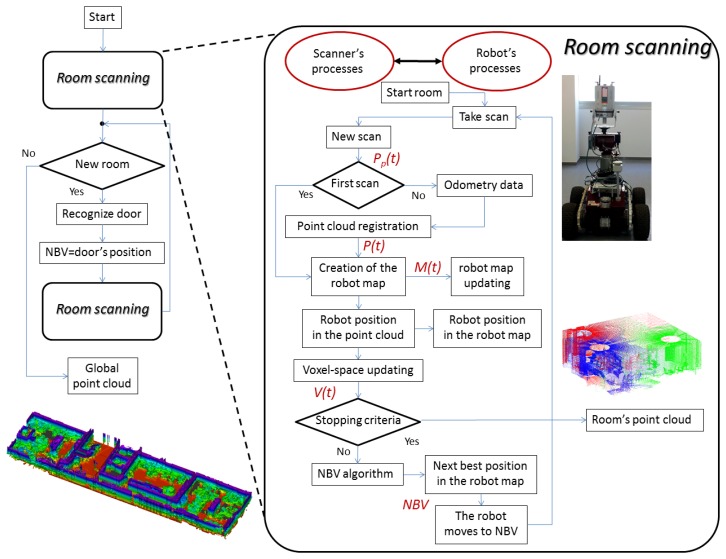
Overview of the automatic scanning system.

Our automatic scanning system is composed of a laser scanner on board a mobile robot. Since the scenario can be composed of several rooms, the system performs the complete scanning of the current room, and then, the robot starts scanning an adjacent room. As we will explain throughout the paper, two stopping criteria determine the scanning completeness of a room. When the scanning finishes, the robot is placed under the door that separates adjacent rooms, and the scanning of a new room begins (see Figure 1 left). The algorithm for detecting open doors in walls is not detailed in this document and can be found in previous papers [28]. From here on, the paper concentrates on explaining the procedure for carrying out the automatic scanning of a single room.

The proposed approach is a cyclic process that begins with a new scan at the current position of the mobile robot in the room. We distinguish two agents, the scanner and the robot, which perform independent, but complementary actions and that communicate with each other during each cycle. In practice, each one processes different algorithms in different computers and transfers the results to each other by using sockets. Thus, sockets provide a secure communication between two different processes on both machines.

Figure 1 (right) shows an outline of the automatic scanning process of a room in which we distinguish the actions carried out by the scanner (on the left) and by the robot (on the right). The communication system follows the typical master-slave paradigm, in which either the scanner or robot can operate as the master or slave.

We also distinguish among two types of information in the cycle. At a certain cycle, *t*, the scanner provides a point cloud, *P_p_*(*t*), and later updates the former point cloud of the scene, thus obtaining *P*(*t*). In other words, the scanner performs the registration of the partial point cloud and the previous global point cloud and obtains *P*(*t*). On the other hand, we have a robot map-based autonomous navigation system. Therefore, the robot uses a certain map to go to the next position. It manages the partial map, *M_p_*(*t*), which is later coupled to the former map of the scene, *M*(*t* − 1), and finally generates *M*(*t*).

For the next cycles, this is used to update the earlier global map *M*(*t* − 1) and to obtain the current map *M*(*t*). This, in turn, allows the scanner to place the robot in the current point cloud and in the current map. Thus, the robot position is precisely set in *M*(*t*). Apart from this, in the next cycles, the next stage consists of obtaining the next best position of the robot with the aim of extracting as much new 3D information as possible. This position is commonly called the next best view (NBV) in the literature.

The NBV stage is the most important in the whole automatic scanning process and will be explained in detail in Section 4. After this, the scanner communicates the NBV to the mobile robot, the robot moves towards the NBV and a new scan is taken. Note that the robot remains motionless until the current map and NBV are available.

This sequence of cycles ends when the system considers that there is enough 3D information to build an accurate and reliable 3D model of the room sensed. At this moment, the laser scanner stops scanning, and the robot goes to the door that separates the adjacent room.

As is explained in Section 1, the automatic scanning process aims to extract a complete 3D dataset of the scene without human intervention. When it comes to an inhabited building, as is our case, the automatic scanning becomes more and more complicated. We assume that the scene may contain unknown and disordered objects, and therefore, we are under clutter and occlusion circumstances. Consequently, some of the essential elements of the scene, say the ceiling, floor and walls, could be occluded by movable objects, like curtains, shelves, lamps, tables or chairs. The next sections are devoted to explaining the main steps and algorithms to achieve an automatic scanning.

## 3. The First Scan

### 3.1. Aligning and Filtering the Indoor Point Cloud

Let us assume an initial state in which nothing is known and the mobile robot is located at an arbitrary position inside an unknown scene. The scanner takes its first scan and collects 3D data from the environment. In our case, the scanner has a field of view of 100°× 360°and takes around five million points per scan. In the end, the information provided is a huge number of 3D coordinates in the scanner coordinate system that needs to be preprocessed in three steps: alignment, filtering and bounding.

The first step consists of aligning the data with a horizontal/vertical coordinate system. Bear in mind that, since the scanner is on board the robot, the scanner coordinate system might not be aligned with the floor's plane. This can occur in practice when the robot stands on a non-uniform ground. Therefore, if we assume that floors lie on horizontal planes and walls lie on vertical ones, an alignment process is necessary.

**Figure 2:**
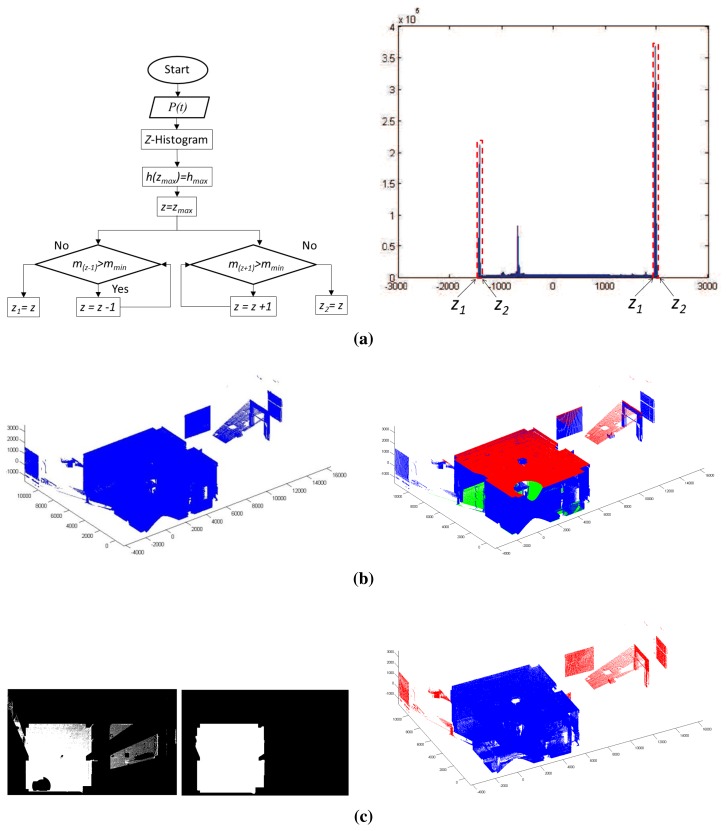
(**a**) Flowchart for obtaining interval [*z*_1_, *z*_2_] and points belonging to the ceiling and floor marked in the Z-histogram; (**b**) original point cloud *P*(*t*) (left) and the points belonging to the ceiling (in red) and floor (in green); (**c**) from left to right: image with ceiling points, extraction of the contour H and the indoor point cloud.

Let us consider XY be the floor's plane. We carry out a brute force algorithm in which the data are rotated according to a small range of Euler's angles. For each rotation, the corresponding Z-histogram is built, and the set of local maximums is identified. The transformation that provides the highest maximum is then chosen as the best one. Figure 2a shows a picture that represents the Z-histogram after the alignment is done. Two local maximums can be seen in this histogram, which correspond to the points belonging to the floor (left) and the ceiling (right). There is another maximum between, which comes from several horizontal tabletops that were also sensed in the scene. In the case of rectangular rooms, an extension of this algorithm can be applied to align vertical walls with planes XZ and YZ.

As is known, when using large range scanners, the captured data might come from outside surfaces. This is owed to the fact that the laser crosses open doors and windowpanes and reaches outdoor objects. Additionally, other outliers come from erroneous data provided by the scanner when the beams reach shiny or reflective surfaces. Therefore, to extract the point cloud of the scene, a data filtering stage has to be done. In our approach, we first delimitate the space belonging to the floor and ceiling, and then, the room's boundaries are calculated.

The points belonging to the floor and ceiling are segmented by analyzing the neighborhood of the first and last maximum in the Z-histogram. Let *h* = *f*(*z*) be the function defined in the histogram, *h_max_* be a generic maximum of *h* and *z_max_* be the center's bin where *h_max_* lies. The algorithm checks the evolution of the slopes *m* of function *h* around *z_max_* after imposing a threshold, *m_min_*. Two bin's centers *z*_1_ and *z*_2_, each at one side of *z_max_*, delimitate the interval in which the threshold is overcome. In such a way, interval [*z*_1_, *z*_2_] allows us to extract the points belonging to the ceiling or to the floor. On the left of Figure 2a, we show the flow chart of this algorithm. On the right, the regions delimitated by the interval [*z*_1_, *z*_2_], for ceiling and floor, are marked in the Z-histogram. Figure 2b (left) represent the original point cloud taken by the scanner and the points belonging to the ceiling (in red) and floor (in green).

The next objective consists of obtaining the room's boundaries in which the mobile robot lies. To do this, we carry out a simple labeling process on the 2D image projected by the ceiling points and take the biggest segment as the one that corresponds to the room's ceiling. After this, the room's contour, H, is calculated over the image that contains these points (see Figure 2c). The room's ceiling points are then defined as the points that fall inside H. Note that, in order to generate the robot map, it is necessary to know which points belong to the ceiling and floor of the room. Therefore, this is an important part of the whole process.

To delimitate precisely the interior of the room, the points belonging to walls are identified as those for which coordinates *x* and *y* are close to the coordinates of the contour H. The extraction of the wall points is carried out following the algorithm of Figure 2a, but in this case, the contour H is recalculated as a sequence of straight segments in the image, each representing the projection of a particular wall. A *d*-histogram over the points close to each straight segment is calculated, *d* being the distance to the segment. Analyzing the maximum in each *d*-histogram, the corresponding points of the wall are obtained. As a result of the earlier stages, the segmentation of the interior points is finally achieved. This is the first point cloud *P*(1) of the scene.

### 3.2. The Flatness Map and the First Robot Map

Sometimes, mobile robots have to navigate through uneven ground and experience small bumps. In our case, these situations should be avoided, because the scanner might fall down when the robot moves towards the next scan position. That is why it is necessary to detect irregular zones in the floor of a scene and to warn the robot of them. These uneven zones are understood in the map that handles the robot (robot map) as obstacles.

The flatness of the scene is studied from the floor's normal vectors map. Since we have a high number of floor points, we group the points into small cells, each containing at least three points, and calculate the normal vectors of these cells. To calculate the normal of a cell, we use the scatter matrix [29] (see Equation (1)) over the points belonging to the cell C. The point closest to the center of C is chosen as the reference point, *p_r_*. Then, the weights of all of the points of the cell, *w*, are calculated according to Equation (2).

(1) $$\begin{array}{cc} M=\sum X^{i^{\tau }}X^{i}\\ X^{i}=\left(w^{i}x^{i},w^{i}y^{i},w^{i}z^{i}\right), & p_{i}\left(x^{i},y^{i},z^{i}\right)\in C \end{array}$$

(2) $$\begin{array}{ccc} w^{i}=\frac{d_{\max}-d^{i}}{d_{\max}-d_{\min}}, & d_{\max}=\max\left\{d\left(p_{r},p_{i}\right),p_{i}\in C\right\}, & d_{\min}=\min\left\{d\left(p_{r},p_{i}\right),p_{i}\in C\right\} \end{array}$$

**Figure 3:**
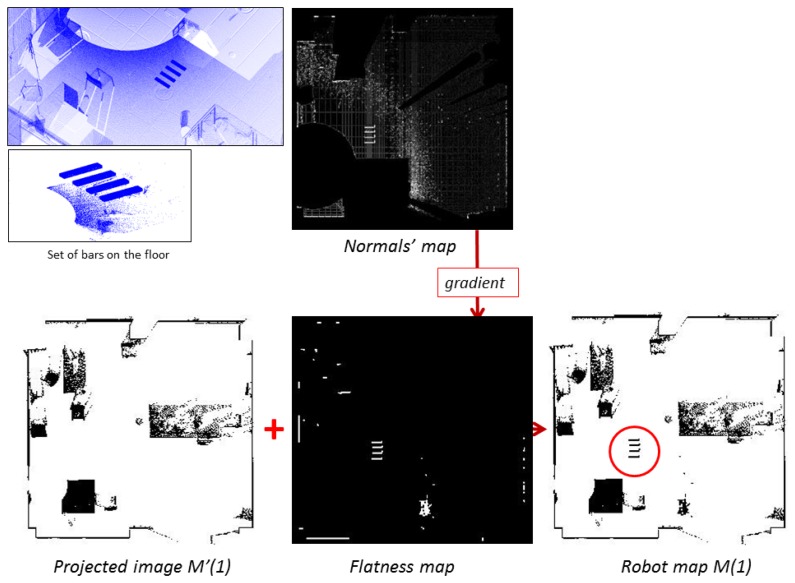
Obtaining the first robot map. (Top) Point cloud with a set of small bars on the floor and the obtained normal's map. (Bottom) Fusion of *M′*(1) and *F*(*1*) and the resulting robot map.

In the scatter matrix, the autovectors that correspond to the highest values define the tangent plane to the cell, and the lowest autovector corresponds to the normal direction. The set of normal vectors can be displayed in an image in which each pixel contains the value of the corresponding angle of the normal vector. The flatness is then analyzed from the gradient of the earlier image. In the gradient image, bright pixels represent high variations of the normal angle, and dark values correspond to flat areas. By imposing a specific threshold in this gradient image, we can obtain a flatness map. Figure 3 (top, left) shows details of a set of small bars on the floor. On the right, a grey image represents the angles of the normal vectors calculated through the scatter matrix's method. The discontinuities introduced by the bars can be seen in the image. The flatness map is shown below.

The first robot map of the scene, denoted by *M*(1), is generated from the point cloud *P*(1) as follows. First of all, ceiling points, floor points and the points above the scanner's height are removed from *P*(1), obtaining *P′*(1). Then, the projection of *P′*(1) is transformed into a 2D image, *M′*(1). Finally, the flatness map, *F*(1), is added, conforming the first robot map *M*(1). This image is understood by the robot as a map in which black pixels mean obstacles. With this map, the robot can accomplish subsequent localization and path planning tasks. Figure 3 illustrates how the first robot map is obtained by fusing *M′*(1) and *F*(1).

## 4. The Next Best Scan

### 4.1. Space Labeling

To calculate the next scanner's position, it is necessary to evaluate the new 3D information obtained from all possible positions of the robot. Since this is an impossible task in a continuous space, the next best view is computed in a discretized space. The discretization factor may influence the runtime and accuracy in subsequent processes. Thus, a coarse discretization may entail false and low-accuracy results, whereas a high discretization factor implies precise results together with a tremendous computational cost. Therefore, a balance of both aspects is mandatory. We need to fix a discretization factor that allows the execution of algorithms in a reasonable period of time, but maintaining the accuracy in the data.

In order to define an environment with an imposed topology over the data, we have built a space composed of voxels, a voxel being a small cube of side *l*. From here on, we will call this space the voxel space. The voxel is, therefore, the unit of a new three-dimensional space, which might contain or might not contain points. We thus create a reduced and discrete space in which it is possible to assign a label to each voxel. Three labels are possible in the voxel space.

- Occupied: This means that the voxel contains at least one point of the point cloud. In this case, the voxel will be an obstacle in the robot map.
- Empty: This signifies that the voxel is seen from the scanner's position, but does not contain any point. Therefore, the voxel will not entail an obstacle.
- Occluded: The voxel is occluded if it is not sensed by the scanner. Since the space of the scene has been calculated in advance, we can state that an occluded voxel is the one that lies between an occupied voxel and the boundaries of the scene. The NBV algorithm's success strongly depends on a good labeling of occluded voxels. Note that the goal here is to lead the robot towards new positions to minimize the number of occluded voxels.

Let V be the current voxel space that contains the scene. V is labeled by ray tracing from the current scanner's position *O*. A generic ray, which goes from *O* to a voxel's center, is used to classify all of the crossed voxels into the three types: occupied, empty and occluded.

Occupied voxels have already been labeled when the voxel space is built. Voxels that are between *O* and an occupied voxel, if any, are labeled as empty voxels. Finally, voxels between an occupied voxel and the scene's boundaries are labeled as occluded voxels.

In order to decide whether the ray collides with a voxel or not, we consider now the voxels as small spheres with radius $r=l/\sqrt{2}$, *l* being the voxel's side. Thus, the ray crosses the voxel if its distance to the voxel's center is less or equal than *r*. Obviously, this discretization of the space may cause errors in the labeling. Note that an intermediate occupied voxel might affect several rays. Particularly, a voxel near the scanner might occlude many others. Consequently, over-occlusion effects can appear, and occupied voxels might become occluded in a ray-tracing process. In the same manner, empty voxels may turn into occluded ones. To avoid these labeling errors, we have defined a ray-tracing procedure for occluded voxels as follows.

**Figure 4:**
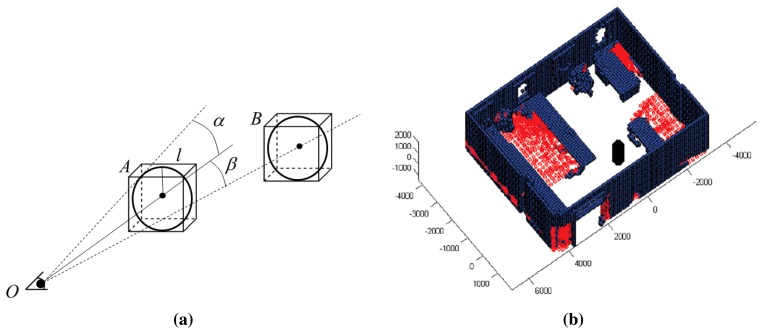
(**a**) Occlusion in the voxel space: Voxel A occludes Voxel B since *α > β*; (**b**) labeled voxel space. The ceiling and floor are removed for a better visualization.

Let us suppose an occlusion case, depicted in Figure 4a. In this example, Voxel B has to be labeled after knowing that Voxel A is occupied. Let *α* be the angle between the A-sphere tangent ray and the ray crossing the A-sphere's center, and let *β* be the angle between rays crossing A-sphere and B-sphere's centers. We state that B is occluded if *α* > *β*; otherwise, B is empty.

Figure 4b shows the results of a labeling process in the scenario of Figure 2. The black cylinder represents here the laser scanner; blue voxels are occupied, and red ones are occluded. The rest of the voxels, that is ‘transparent voxels’, are empty. Note that the voxels belonging to the ceiling and floor have been removed for a better visualization.

### 4.2. Next Best View

The next best position of the scanner (or mobile robot) is calculated in the voxel space and then is translated to the robot map. Basically, we estimate the amount of occluded voxels that would turn into occupied or empty voxels from other scanner position.

The labeled voxel space below the scanner's height, *V_s_*, is synthesized into a 2D labeled image, *lxl* being the size of the pixel. From now on, we will name this image the “labeled map”. The labeled map is just the results of a particular label projection procedure. We carry out a top projection of the labels that are below the scanner's height and store them in the corresponding pixel. This means that more than one label might fall in the same pixel. In such a case, only one label is assigned to the pixel after following a priority order. The priority rule is as follows: first, choose occupied, then occluded and, finally, empty. Obviously, the robot should navigate in a collision-free area, and consequently, the highest priority should correspond to occupied voxels. It is also clear that if occluded and empty labels fall into the same pixel, the pixel should keep the occluded label, because the objective of the algorithm is indeed to increase the information of the scene.

The next best position in the labeled map's context must verify three requirements, which have to do with labeling, security and path planning:

1. Label requirement: the pixel has to be labeled as empty.
2. Security requirement: the distance from the pixel to any occupied pixel has to be higher than certain security distance; this requirement is imposed in order to guaranty the secure moving of the robot in the scene.
3. Path planning requirement: there is at least one path from the current position of the robot to the candidate pixel that meets Conditions 1 and 2 for each pixel of the path.

**Figure 5:**
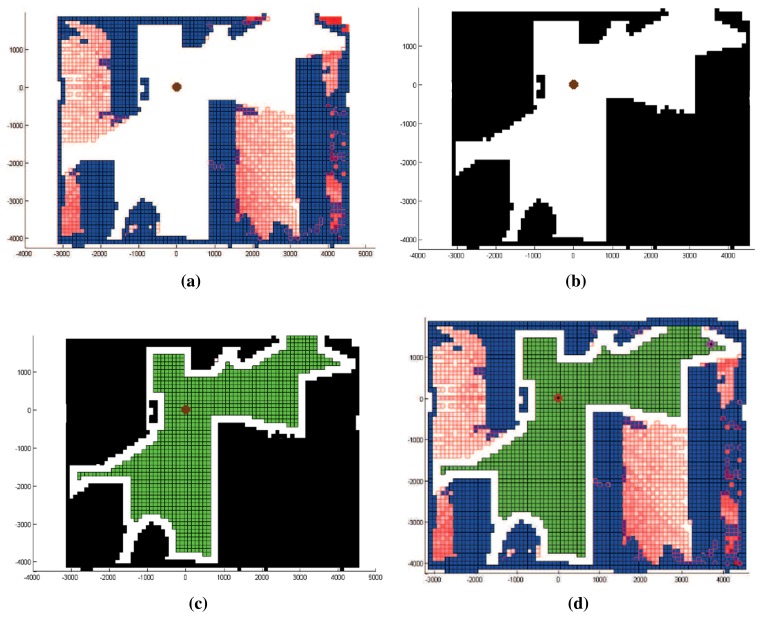
(**a**) Initial labeled map; (**b,c**) permitted and non-permitted positions before and after imposing security and path planning requirements; (**d**) final labeled map.

Figure 5a shows the labeled map obtained from the example of Figure 4. Occluded regions are painted in red, and occupied ones are in blue. Permitted and non-permitted next positions, before applying Requirements 2 and 3, are illustrated in Figure 5b. Here, the black color signifies non-permitted positions, and the white color means possible next positions. When security and path planning requirements are imposed, the final labeled map is obtained. In Figure 5c, we represent the security region in white and the next best view area in green. The final labeled map, including the security regions, is shown in Figure 5d.

In this framework, the goal of our algorithm is to maximize the number of occluded pixels that change their label from the next view point. This implies that some earlier unknown zones might now be seen and be registered in an updated labeled map. We assume that occupied and empty pixels keep their state and that only occluded pixels may change.

**Figure 6:**
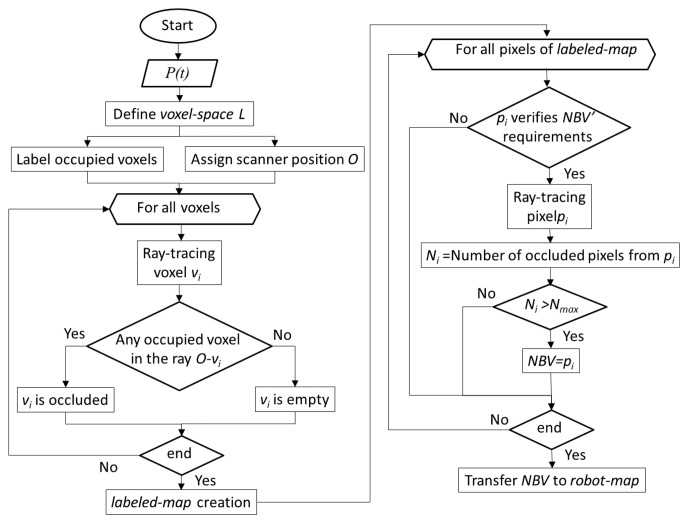
Chart containing the labeled map creation and the next best view (NBV) algorithm.

The flowchart of the space labeling and the NBV algorithm is plotted in Figure 6. A ray tracing is executed from the candidate positions that verify the requirements, and the number of occluded voxels seen from each position is stored. The position from which the maximum number of occluded pixels is viewed is taken as the next best view. Note that the NBV must verify Requirement 2, and consequently, there exists a path from the current to the next position of the robot. The NBV is finally inserted in the robot map.

**Figure 7:**
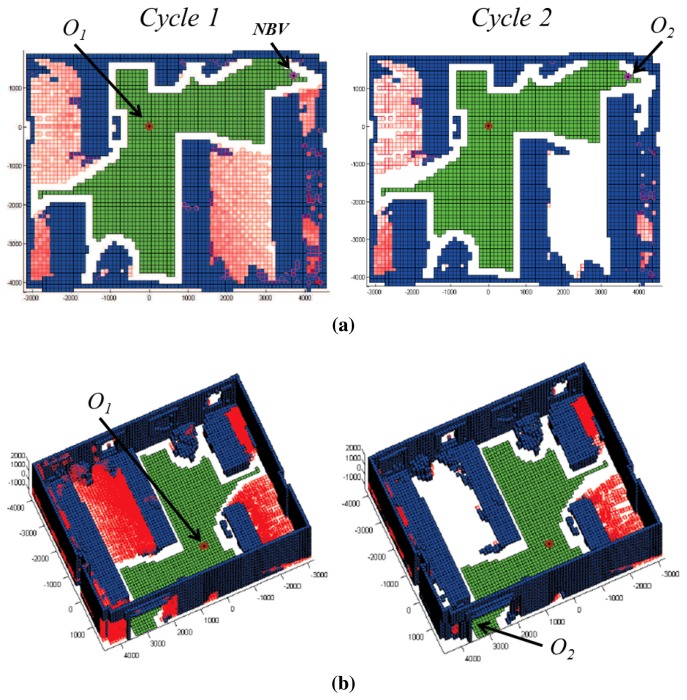
(**a**) Labeled map for Cycles 1 and 2 after applying the NBV algorithm; (**b**) labeled voxel space for Cycles 1 and 2.

Figure 7a shows the results achieved after applying the NBV algorithm. The new position of the scanner is marked in the earlier labeled map, and the updated labeled map is shown on the right. Note that some red pixels (occluded) have turned into white pixels (empty), and consequently, the entropy of the scene has increased. Figure 7b shows the three-dimensional voxel space in which some regions (pointed out in the figure) are relabeled as occupied voxels.

## 5. The Following Scans

At the moment, the robot is placed in the new position (at the beginning of a cycle *t*), and the system has gotten the previous point cloud *P*(*t*−1), robot map *M*(*t*−1) and labeled voxel space *V*(*t*−1). When the robot orders a new scan, the odometry data are sent to the scanner. Afterwards, a new partial point cloud *P_p_*(*t*) is obtained, and the subsequent stages are run again. In the first stage, the scanner carries out the alignment of the partial point cloud *P_p_*(*t*) in the global coordinate system. Through the odometry data, a coarse transformation is first calculated, and then, a precise registration of *P_p_*(*t*) and *P*(*t* − 1) is carried out, obtaining *P*(*t*). The registration is accomplished applying the well-known iterative closest point technique [30].

**Figure 8:**
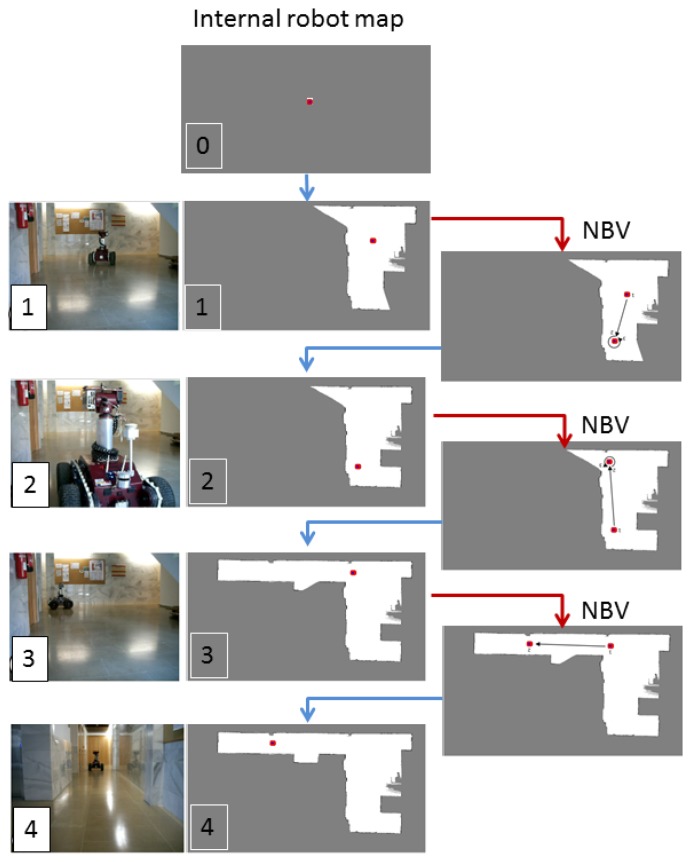
Mobile robot navigation in a corridor. The internal map used by the robot in each cycle is shown.

The subsequent stages were already explained in Section 2. The scanner generates a new robot map *M*(*t*) and sets the robot's position on it. This information is transferred to the robot. Then, the newly-labeled voxel map *V*(*t*) is calculated; the scanner runs the NBV algorithm again and sends the new position to the robot. Figure 8 shows the evolution of the map that the robot manages during the automatic acquisition of a corridor. We considered that the surface of the scene was fully sensed after four scans.

Scanning approaches usually apply stopping criteria depending on their own parameters and assessment functions [11]. Devy *et al.* finish the scanning process when the scanner cannot reach unseen areas. Besides, they impose a certain quality parameter on the occupied voxels [9]. In [14], the size of the borderline area between empty and unknown regions is evaluated to establish the stopping criteria. For their part, Blaer *et al.* take into account the number of borderline voxels, that is unknown voxels with an empty neighbor [16]. Lopez *et al.* [10] use, among other criteria, a limit for the number of the so-called occplane voxels (occluded voxel with at least one empty neighbor).

In our case, two stopping criteria determine the scanning completeness of the room. The stop criteria are established as a function of the labeled voxel space's growing rate, λ, and the occluded voxel's decreasing rate, *μ*. These are empirical parameters, which depend on the scenario's size, and are calculated according to Equations (3) and (4), *V*(*t*) being the number of occupied and empty voxels in the cycle *t, V_s_* the total number of seen voxels, *O_c_* the number of occluded voxels in the cycle *t* and *V* the voxel space size. In our case, we require values λ < 0.04 and *μ* < 0.05 for stopping the scanning process in the room. As mentioned in Section 2, the stopping criteria are applied for scanning a single room. Therefore, when the room has been scanned, the robot then moves towards the door that separates an adjacent room, and the scanning of this room begins.

(3) $$\lambda =\frac{\Delta V\left(t\right)}{V_{s}}$$

(4) $$\mu =\frac{-\Delta O_{c}\left(t\right)}{V}$$

## 6. Experimental Results

The method presented in this paper has been tested in dense point clouds of inhabited interiors. The experimental setup is composed of a Robotnik Guardian mobile robot that carries a Riegl VZ-400 laser scanner. Inside the robot, two computers execute processes concerning the scanner and the robot. The processing time is mostly devoted to 3D data processing tasks (95%) and robot movements (5%). The scanner collects around five million points in 45 s and takes less than 6 s for registration tasks. The NBV algorithm is calculated in 39 s. To carry out the discretization and labeling of the space, voxels of a 10-cm side were taken.

**Figure 9:**
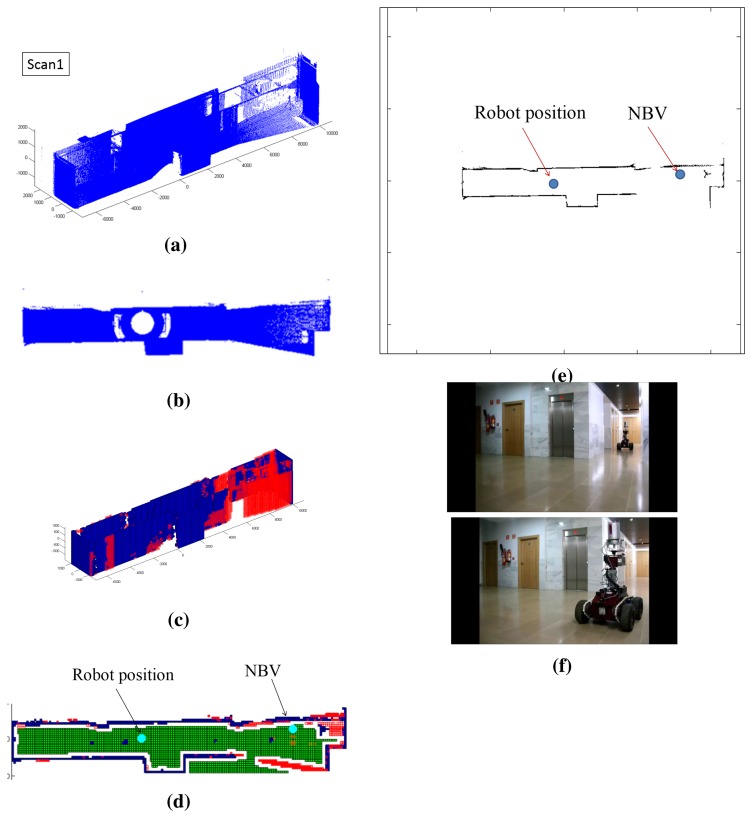
Results in the first scan. (**a**) Initial point cloud; (**b**) points belonging to the ceiling; (**c**) the first labeled voxel space *V*(1); (**d**) position of the next best view in the labeled map; (**e**) robot map and the next position; (**f**) pictures of the mobile robot in Positions 1 and 2.

**Figure 10:**
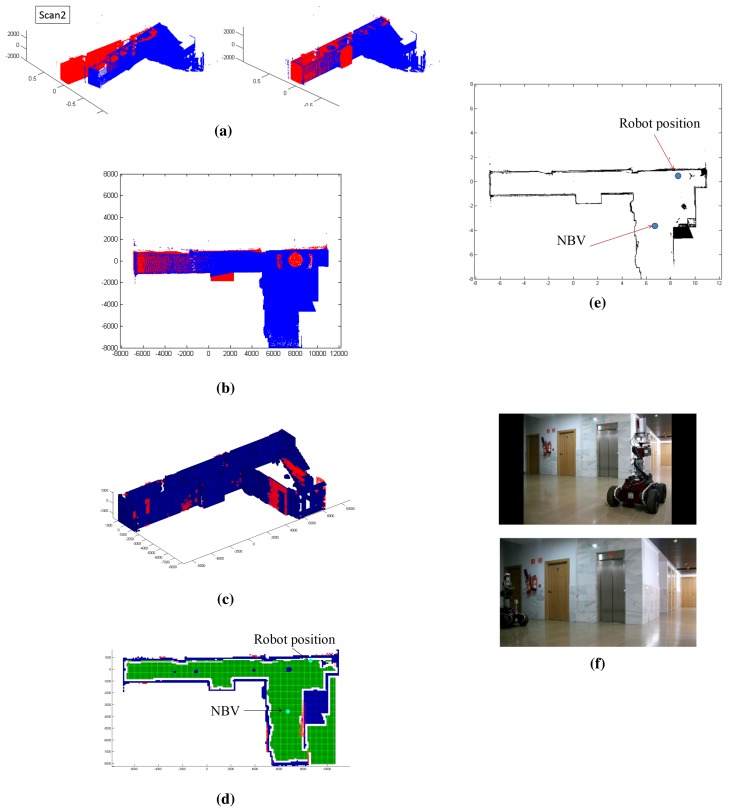
Results in the second scan. (**a**) Registration stage; (**b**) points belonging to the ceiling; (**c**) the second labeled voxel space *V*(2); (**d**) position of the next best view into the labeled map; (**e**) robot map and the next position; (**f**) pictures of the mobile robot in Positions 2 and 3.

The test has been carried out on the first story of a high school in which the flatness of the floor is almost absolute. Only a few small irregular zones have been placed on the floor of the scene. Anyway, the stability during scanning is not affected, because the scanner only takes scans when the robot is standing still. In order to guarantee the safe movement of the whole system, when the robot moves, the stability of the platform is regulated under a PID control algorithm. Figures 9 and 10 illustrate the outputs of some of the stages of the whole process for the first and second scans. In this case, the scene corresponds to a part of the corridor with no obstacles. Figures 9a,b show the first point cloud *P*(1) and the points belonging to the ceiling. Figures 9c,d illustrate the first labeling of voxels and the labeled map with the next best view. Figure 9e represents the robot map *M*(1), which includes the current and next position of the mobile robot. In Figure 9f, there are pictures of the mobile robot in both positions. Figure 10 corresponds to the second scan. As can be seen in Figures 10a,b, the new point cloud *P_p_*(2) (in blue) is registered with the first one *P*(1) (in red). Thus, the point cloud of the scene is updated, obtaining *P*(2). The rest of this figure shows the results of the following stages: labeling, NBV and the updated robot map.

**Figure 11:**
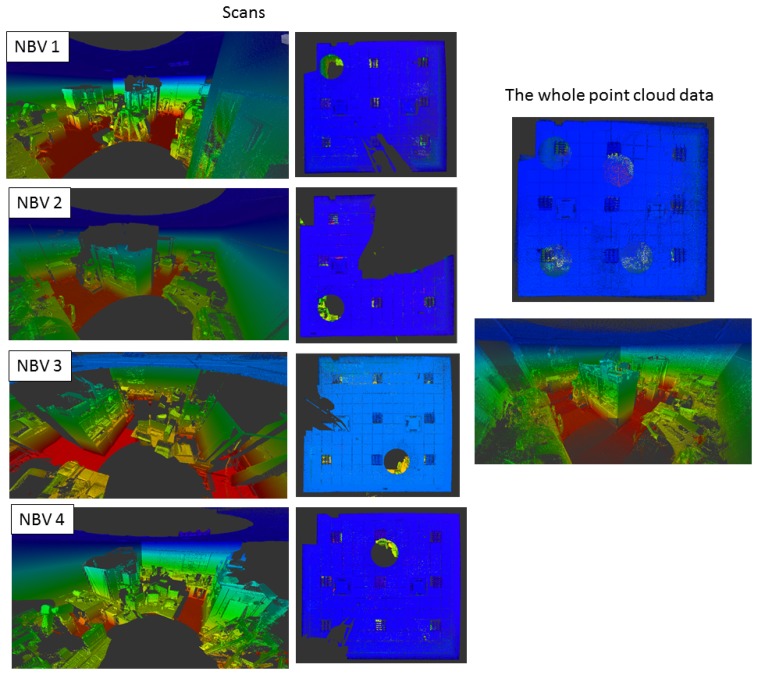
Scanning of Room Number 1 after four robot positions. (**Left**) Panoramic and top view of the each point cloud; (**right**) the whole point cloud generated.

Figure 11 shows details for one of the rooms of the story. Four scans were taken to complete the scanning in a complex scene with several desks, chairs, bookshelves and other objects. On the left, the point cloud for each position is represented in a color code. Furthermore, a top view is provided. Note that the position of the scanner can be clearly seen by the gaps that appear from the top view. On the right, the whole point cloud is shown.

Table 1 provides some data that concern the 3D processing and NBV stages. We include the number of scans *N*, the number of points per room *P* and the number of seen voxels *V_s_*. The labeled voxel space's growing rate λ is shown in the next row. We also give information about the efficiency of the NBV algorithm. The number of each type of voxel, occupied (*O*), empty (*E*) and occluded (*O_c_*), is included in the following rows. It is interesting to analyze the evolution of percentages *O/V*, *E/V* and *O_c_/V*, *V* being the voxel space size. Note the increment of the voxel space also determines the end of the process according to parameter *μ*. In our case, the algorithm finishes when the occluded voxel's decreasing rate is below 0.05 and the labeled voxel space's growing rate is below 0.04.

In order to assess the goodness of the results and to make comparisons, we have built by hand the geometrical ground truth of the scene. The ground truth model was carried out with the help of a Leica DISTOTM A6 laser tape measure, which provides one-millimeter accuracy.

**Figure 12:**
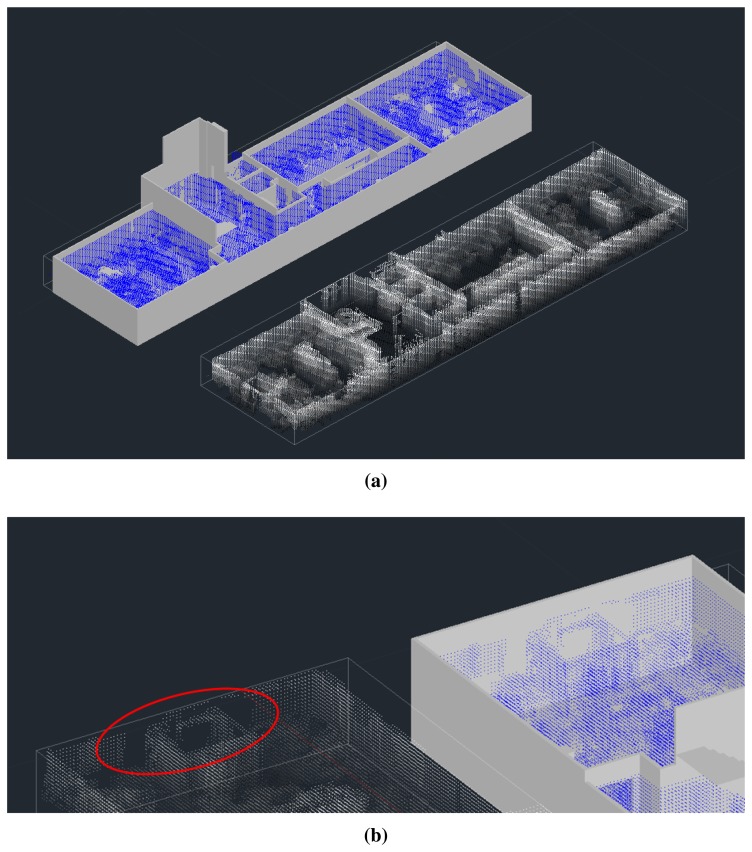
(**a**) Global point cloud superimposed on the CAD model of the first floor of the building (top) and the image of the point cloud alone (the ceiling has been removed in both); (**b**) details of unscanned areas.

Figure 12 presents the final point clouds of the first floor superimposed on the ground truth model. We measured the percentage of the surface of the structural elements of the building, that is floor, ceiling and wall areas, that have been sensed. Table 2 provides a summary of the results per room and per item, including absolute areas (in m^2^) and relative data with respect to the ground truth. The percentage of the total scanned area was 80.4%, which means that 19.6% of these elements was occluded by furniture or other objects at the scanning time. Non-sensed zones can come from several sources, occlusions and scanner errors being the most important ones. As can be seen in Figure 12b, some pieces of furniture occlude floor and walls. Additionally, the scanner is unable to reach some zones of the scene, and consequently, several parts of the building might be missed in the global point cloud. Anyway, we will be able to reconstruct the total structure of the building in further modeling stages.

Therefore, the next objective for a complete reconstruction of the indoors would be to convert this raw information into a high-level surface representation, which includes the essential elements in construction, that is the ceiling, floor and walls. Within the 3D representation models universe, we have chosen the boundary representation (B-rep) model [31]. In a B-rep representation, a shape is described by a set of patches, edges and vertices along with their connectivity. Basically, we start segmenting the data belonging to the ceiling, floor and walls; we then calculate the planes that best fit every set of segments, and finally, the intersections between connected planes are performed. As mentioned in Section 1, the creation of 3D building models is not the objective of this paper. Information about algorithms that generate B-rep models from point clouds can be found in earlier papers [28,32].

## 7. Conclusions and Contributions

In recent years, 3D measurement systems have become increasingly popular in civil engineering and construction. These systems have been handled for years by an operator for sensing and modeling facilities and large scenarios. Particularly, in the data acquisition stage, the scanner operator usually decides the number and locations of the scanner, which makes this process highly inefficient and time consuming. Besides, the quality and the information of the scene are frequently incomplete. Therefore, optimizing this process will yield evident advantages in the 3D digitization field.

With this motivation, the objective of this paper was addressed towards intelligent and automatic 3D data acquisition. We present here a methodology to carry out a complete scanning process of the interiors of buildings using a 3D laser scanner on board a mobile robot.

To date, most of the works related to automatic scanning solely proposed solutions related to the next best view problem, many of them in simulated conditions and from a 3D-data-processing point of view. In other words, it is supposed that the 3D data are available in advance and that the NBV algorithm can be applied to them. Then, the algorithm's output is provided to the operator, who is supposed to be able to move the scanner to the next position. This makes such proposals just partial solutions of the general automatic scanning problem.

A scarce number of authors deals with automatic scanning with robots, and very few of them reconstruct indoors with the detail achieved in our developments. That makes this field a current and encouraging topic, in which much research has to be done.

The main contribution of this work lies in that we propose a strategy that automates the indoor scanning process in a complete sense with the help of mobile robots. Our system considers two agents, the scanner and the mobile robot, and their interaction in the process. The method provides 3D processing solutions, *i.e.*, the NBV algorithm and robot map generation. Moreover, the system is able to autonomously navigate in scenarios of medium complexity, as is a story of a building.

A lot of work has to be done in the future with respect to our current proposal, and many new ideas need to be developed in the future. To start with, some important aspects related to the updating of the robot's operating system and the implementation of a faster NBV algorithm need to be sorted out. Another point to be tackled in the future is implementing an algorithm for selective scanning. This would allow us to avoid data redundancy and, thus, greatly reduce the point clouds, making the algorithms presented in this paper easier and less time consuming. Finally, we are planning for the next few years a system that will be able to carry out complete scanning of buildings for several floors. This entails generating new scene understanding algorithms on dense point clouds and also more complex robot control tasks.

## Figures and Tables

**Table 1:** Results concerning 3D processing and NBV stages.

**Room**	**1**	**2**	**3**	**4**	**5**	**6**
*N*	4	5	1	1	3	4
*P*	21,673,366	22,747,438	4,807,222	5,207,355	1,562,761	20,723,915
*V_s_*	185,927	185,391	11,026	9757	149,029	195,105
*λ*	0.039	0.025	-	-	0.037	0.036
*O*	45,135	40,479	2875	2261	33,825	42,292
*E*	140,792	144,912	8151	7496	115,204	152,813
*O_c_*	25,273	10,143	8151	7496	115,204	152,813
*O/V*	0.214	0.207	0.216	0.170	0.201	0.196
*E/V*	0.667	0.741	0.613	0.564	0.684	0.707
*O_c_/V*	0.120	0.052	0.171	0.266	0.115	0.098
*μ*	0.034	0.041	-	-	0.032	0.033

**Table 2:** Results of the sensed areas.

**Room**	**1**	**2**	**3**	**4**	**5**	**6**	**TOTAL**	**Pct (%)**
**Ground Truth Walls (m^2^)**	96.60	153.20	21.92	23.44	87.60	94.20	476.96	-
**Detected Walls (m^2^)**	68.40	140.72	15.32	16.36	74.24	69.36	384.40	80.6
**Ground Truth Floor (m^2^)**	68.80	48.16	4.80	3.84	72.16	45.76	243.52	-
**Detected Floor (m^2^)**	29.84	47.40	3.20	1.28	41.88	30.84	154.44	63.4
**Ground Truth Ceiling (m^2^)**	68.80	48.16	4.80	3.84	72.16	45.76	243.52	-
**Detected Ceiling (m^2^)**	68.76	43.16	3.92	2.92	72.16	45.76	236.68	97.2
**TOTAL Ground Truth (m^2^)**	234.20	249.52	31.52	31.12	231.92	185.72	964	-
**TOTAL Detected (m^2^)**	167	231.28	22.44	20.56	188.28	145.96	775.52	80.4
**Percentage (%)**	71.3	92.7	71.2	66.1	81.2	78.6	80.4	-
